# What Does That Head Tilt Mean? Brain Lateralization and Sex Differences in the Processing of Familiar Human Speech by Domestic Dogs

**DOI:** 10.3390/ani15213179

**Published:** 2025-10-31

**Authors:** Colleen Buckley, Courtney L. Sexton, George Martvel, Erin E. Hecht, Brenda J. Bradley, Anna Zamansky, Francys Subiaul

**Affiliations:** 1 Center for the Advanced Study of Human Paleobiology, Department of Anthropology, The George Washington University, Washington, DC 20007, USA; colleen_buckley@gwmail.gwu.edu (C.B.); subiaul@gwu.edu (F.S.); 2 Department of Population Health Sciences, Virginia-Maryland College of Veterinary Medicine, Virginia Polytechnic Institute and State University, Blacksburg, VA 24061, USA; 3 Department of Information Systems, University of Haifa, Haifa 31905, Israel; 4 Department of Human Evolutionary Biology, Harvard University, Cambridge, MA 02138, USA; 5 Department of Speech, Language and Hearing Sciences, The George Washington University, Washington, DC 20007, USA

**Keywords:** dog cognition, language processing, social communication, interspecies communication

## Abstract

**Simple Summary:**

Dogs display many behaviors and expressions when interacting with human companions. Among these behaviors, people frequently observe dogs tilting their heads in one direction or the other when they are being spoken to. Despite being a commonly observed behavior, the origin and purpose of head-tilting in dogs is not well understood. In this study we use the DogFACS coding system coupled with AI analyses to review video recordings of household dogs responding to communication from their human owners. We examine head tilts to try to determine when and how dogs exhibit this behavior, and if it may be related to language processing. We find that communicative cues from people elicit more head tilting from dogs, and that there may be sex differences related to tilt frequency and directionality. Our findings have important implications for understanding human–dog interactions and language processing in non-human animals.

**Abstract:**

Does the head tilt observed in many domesticated dogs index lateralized language processing? To answer this question, the present study evaluated household dogs responding to four conditions in which owners provided an increasing number of communicative cues. These cues ranged from no communicative/affective cues to rich affective cues coupled with dog-directed speech. Dogs’ facial responses were first coded manually using the Dog Facial Action Coding System (DogFACS), followed by an in-depth investigation of head tilt behavior, in which AI-based automated analysis of head tilt and audio analysis of acoustic features extracted from communicative cues were implemented. In a sample of 103 dogs representing seven breed groups and mixed-breed dogs, we found significant differences in the number of head tilts occurring between conditions, with the most communicative (last) condition eliciting the most head tilts. There were also significant differences in the direction of the head tilts and between sex groups. Dogs were more likely to tilt their heads to the right, and neutered male dogs were more likely to tilt their heads than spayed female dogs. The right-tilt bias is consistent with left-hemisphere language processing in humans, with males processing language in a more lateralized manner, and females processing language more bilaterally—a pattern also observed in humans. Understanding the canine brain is important to both evolutionary research through a comparative lens, and in understanding our interspecies relationship.

## 1. Introduction

The head tilt behavior is ubiquitous in popular representations of dogs. But why dogs engage in this behavior remains unclear and attempts to explain it are sparse in the literature. Given the many communication-related adaptations dogs have developed in the context of the human–dog relationship [1,2], some have hypothesized the head tilt may serve a communicative function in interspecies interactions, that is, to elicit positive affect among human observers. In one study, for example, human participants who were asked to modify photos of puppies to make them appear “cutest” consistently manipulated photos to show the puppy with a head tilt [3]. A follow-up study asked participants to judge the “cuteness” of head tilts, finding that both puppies and adult dogs were deemed “cuter” when pictured as tilting their heads [4]. The authors suggest that the participants’ responses may inform welfare-related activity, such that understanding the effect of the head tilt behavior could enable animal shelter staff to make their dogs and puppies more marketable for adoption (e.g., in photographs).

Another area of research providing some further information on the behavior has explored whether the head tilt response serves as a type of cognitive offloading in dogs. In humans, cognitive offloading occurs when we use physical actions to decrease the cognitive demand of processing stimuli, such as tilting our heads to process a slanted image or gesturing to help us picture objects that are not physically present [5]. One study showed that while some dogs tilt their heads to follow the direction of a rotated visual stimulus, the head tilts did not affect their ability to recognize rotated stimuli [6]. This result contradicts the hypothesis that head tilts function as a cognitive offloading strategy for dogs when processing visual stimuli.

Thus, given the inconsistencies in the available literature regarding the purpose of the head tilt in dogs, we are left with many questions about the behavior’s functionality, as an adaptation or otherwise. One potential line of questioning that has not been explored to a great degree in dogs but that could offer additional insight relates to brain lateralization.

Lateralized bodily behaviors such as hand preferences or head turns have been used as proxies for understanding brain lateralization for over a century [7,8,9]. While the lateralized process of human speech has not been extensively explored in dogs, there is evidence for lateralization in the dog brain. When processing visual stimuli, dogs were shown to have lateral biases when presented with photos of human faces depicting different emotions [10]. The head turn behavior is an asymmetrical movement already associated with lateralized brain function [11,12]. In head turn studies, dogs show significant left-hemispheric bias for processing familiar language that lacked intonation, but familiar words with positive intonation did not elicit significant differences in the head turn bias [11]. The head tilt—a type of head turn—behavior is another asymmetrical behavior that has received relatively less empirical attention. To the best of our knowledge, only one study has provided a description of the head tilt behavior in dogs and linked it to speech processing [13]. The authors utilized an object-label knowledge test, examining if dogs that were able to learn the names of specific objects tilted their heads differently than those who did not. The dogs who were able to learn object names tilted their heads more frequently than those who did not, suggesting a relationship with processing relevant stimuli. However, that study had a limited sample size and examined the head tilt behavior in a limited context rather than using naturalistic speech, limiting its generalizability.

The head tilt behavior as an index for lateralized human speech processing has not been well-explored using sufficient sample sizes and breeds. To fill this gap, we performed an in-depth investigation of head tilt behavior using both manual behavioral coding and AI-based automated analysis to measure head tilt. The use of AI is an important emerging technology in studies of animal behavior, and we have incorporated these methods here in an effort to add to the growing body of literature exploring the efficacy of such tools, especially in comparison to manual coding [14,15,16,17]. In addition, we extracted and analyzed acoustic features from audio signals of the communicative cues. Using these measurement tools, we examined the occurrence, direction, and amplitude of the head tilt behavior when pet dogs are presented with both familiar and unfamiliar words, while also looking at the acoustic features of the communicative cues. Specifically, we evaluated whether the head tilt behavior was lateralized and consistent with left-hemisphere lexical processing or a stable but idiosyncratic (non-lateralized) behavioral response.

## 2. Materials and Methods

### 2.1. Subjects

A total of 103 dogs were included in this study (Range 0.5–14 years, Mean age = 5.2, SD = 3.2; F = 53). Data were collected on five additional dogs, but they were not used in data analysis due to poor video quality. The final sample comprised 53 female dogs (5 intact, 48 spayed) and 50 male dogs (10 intact, 40 neutered). Dogs were grouped into age brackets: Young—6 mo to 2 years, (*n* = 20); adult—2.1–6.9, (*n* = 49); and senior—7+ years, (*n* = 34) [18]. Dogs were also grouped into one of the seven groups recognized by the American Kennel Club [19], plus mixed-breed dogs. See Supplementary Materials for details.

### 2.2. Procedure

The data utilized in this study is part of a data set collected for a larger project on canine facial communication [20].

Due to the COVID-19 pandemic, most data were collected via community science, using owner-submitted videos between November 2020 and February 2022. Dogs and owners were recruited via social media and outreach directed at dog owners. Owners were entered into a drawing for a Chewy.com gift card and received a certificate of participation. See Sexton et al. [20] for additional data collection details and protocol details. Briefly, owners were instructed to use their cell phone cameras to produce four 30 s videos and keep their dog’s face fully visible throughout. All four videos were to be completed within 72 h, allowing at least 30 min between recordings and conditions.

All dogs participated in four communicative conditions, consisting of 1 trial each, were presented in serial order (not counterbalanced), with each condition including more communicative cues than the preceding condition:

Condition 1: Served as baseline measure with the dog at rest and the human making no eye contact.

Condition 2: Human makes eye contact with dog, no speaking or gestures.

Condition 3: Human makes eye contact with dog, repeats unfamiliar phrase, twice, slowly: “Ancient Egyptians built enormous pyramids to honor the pharaohs. Ruins from many of these sites have been excavated over the years, unearthing mummies, art and relics.”

Condition 4: Human makes eye contact with the dog, speaking in a normal-pleasant tone, using words or phrases familiar to the dog (idiosyncratic and unscripted).

Each condition consisted of a single video unless the dog’s face moved out of frame, in which case they were to be re-filmed. We did not require owners to indicate whether re-filming was necessary in order for them to achieve a suitable video, and we do not have data reflecting how often re-filming was necessary. Owners ultimately uploaded only one video for each condition.

Per the provided study protocol and instructions, participants were asked to locate a quiet, well-lit area of the home to conduct the recording sessions and, where possible, to avoid distractions, such as other humans, dogs, animals, etc. All videos included in the final analysis observed these general instructions, though due to the nature of community science and at-home data collection, participant positioning and environment varied somewhat across participants. In keeping the dogs’ faces visible in each video, most participants stood or sat in front of their dogs.

### 2.3. Behavioral Coding

**Figure 1:**
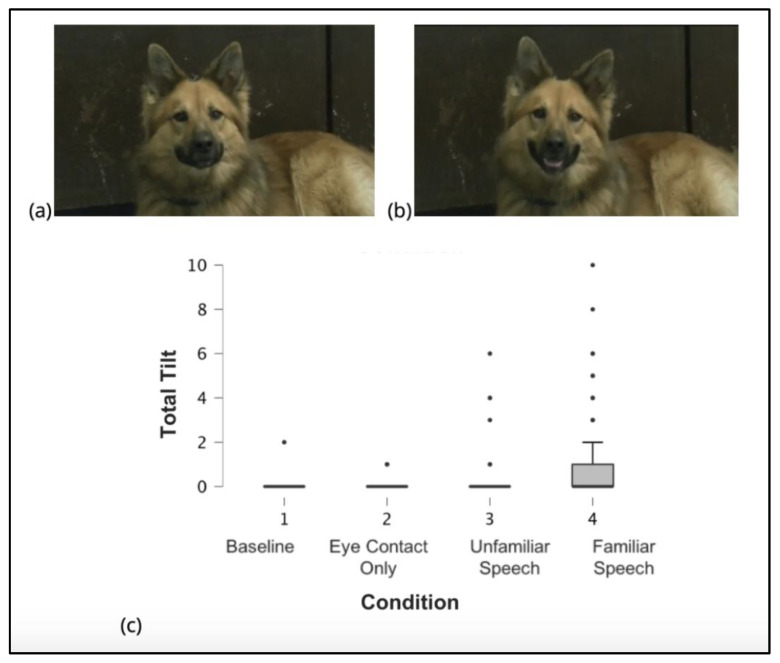
The head tilt behavior depicted in photos and as seen across conditions. (**a**) Dog with head in a neutral position. (**b**) Dog illustrating the head tilt (leftwards), as seen in the DogFACS manual by Waller et al. [21]. (**c**) Mean and standard deviation of head tilt occurrences in each condition.

Dog Facial Action Coding System (DogFACS) was used to identify and categorize all facial movements in dogs in a standardized fashion [21]. Two certified DogFACS coders coded the frequency and duration of all action units included in the DogFACS manual. Coding was limited to the 30 s video duration for each condition. The only action descriptors included and analyzed in the present study are head tilt left (AD 55) and/or head tilt right (AD 56) responses. The certified coders independently reported head tilt left (AD 55) and/or head tilt right (AD 56) responses. Head tilt was coded when the ear on the associated side of the body moved down toward the floor. Figure 1a,b illustrate the dog head tilt.

### 2.4. Inter-Rater Reliability

As was noted above, all videos were coded independently by two separate DogFACS-certified coders. For dogs whose intercoder behavioral scores were different by more than 5 points, videos were rescored. No videos needed to be discarded due to coder discordance.

### 2.5. Artificial Intelligence (AI)-Based Head Tilt Measurement

**Figure 2:**
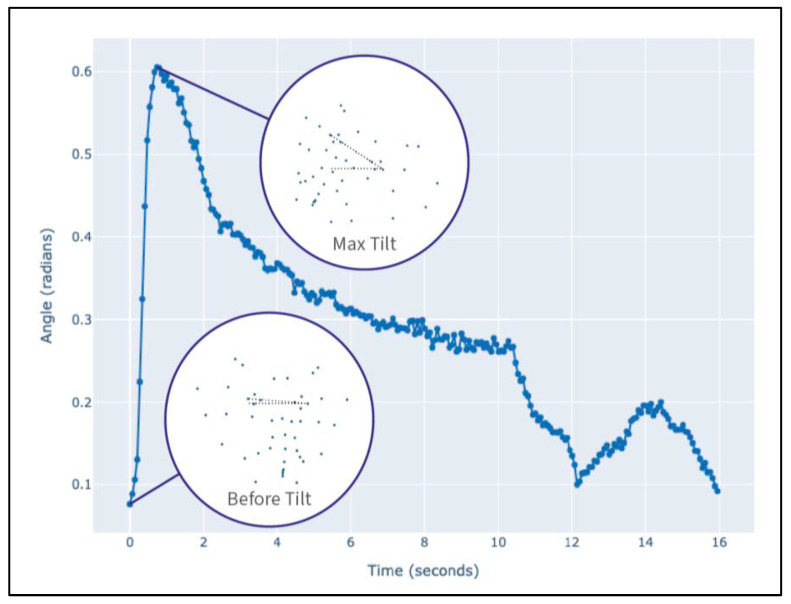
The head tilt angle change during the random video from the dataset. The scatterplots in circles demonstrate the 46 landmarks on the dog’s face in two moments—before the tilt and at its peak. The tilt angle is measured as their difference.

To quantify the head tilt, we utilized the Ensemble Landmark Detector (ELD) [22], trained on the DogFLW dataset [23], which detects 46 facial landmarks grounded in DogFACS action units. For head tilt amplitude measurement, we extracted two frames using the manually coded head tilt labels: head tilt start and head tilt peak. Using these two frames for each head tilt, we measured the difference between the angles between the *x*-axis and the line connecting outer eye corners. Figure 2 illustrates the change in tilt angle between the two frames.

### 2.6. Audio Feature Extraction and Analysis

We processed the audio signals of the communicative cues in Condition 4 for testing their correlation with the head tilt’s direction or amplitude. To do this, for each coded tilt, we divided the communicative cues into four categories based on the word’s meaning: *Activity*, *Addressing*, *Food*, and *Toy*. The *Activity* category included words related to various activities primarily associated with walking, such as ‘walk’, ‘outside’, ‘out’, ‘go’, etc. The *Addressing* category encompassed calling dogs by name and words such as ‘boy’, ‘girl’, ‘dog’, ‘[what are you] doing’, etc. The *Food* category included owners mentioning various meals, as well as the words ‘treat’ and ‘hungry’. The *Toy* category contained the words ‘toy’, ‘ball’, and other toys’ names.

We then extracted from the audio signals volume and pitch parameters. Volume was quantified as Root Mean Square (RMS) energy using the librosa Python library, which computes the energy of the audio signal in overlapping frames (2048 samples per frame, 512 hop length). RMS values were mapped to corresponding time points to analyze loudness variations over time. The pitch was estimated using the probabilistic YIN (pYIN) algorithm in the librosa library, which calculates the fundamental frequency in voiced segments within a frequency range of 65.41 Hz to 2093 Hz. Missing values for unvoiced segments were interpolated to ensure smooth visualization. For statistical analysis, we normalized volume and pitch values for all videos and used the local maximums of segments.

### 2.7. Statistical Analyses

Statistical analyses for DogFACS behavioral codes were performed using JASP [Version 0.17.1] and for audio features using Statsmodels in Python, Version 0.14.4. A generalized linear mixed-effects model (Poisson family, identity link due to low means and many zero counts) was used to test the effect of Condition on head-tilt frequency, with Subject included as a random intercept to account for repeated observations within individuals. Two separate models explored whether demographic variables (e.g., origin, breed group, time in home, age, and sex) predicted variation in head-tilt behavior. Chi-squared tests were used to explore whether head-tilt direction differed by sex and reproductive status.

### 2.8. Data Availability

All data used to generate figures and perform analyses can be found at: https://osf.io/ypsw2/?view_only=7fbe404e20d64328933dc807b7ac7b84. Video data may be available upon request in accordance with privacy considerations. Subject coding data from the parent study are available publicly via Mendeley Data: doi:10.17632/br92x9768y.1.

## 3. Results

### 3.1. Head Tilt and Age, Breed, Sex, and Time Lived in the Home

Forty-one dogs of 103 in this study (40%) performed a head tilt in any condition. A Poisson mixed-effects model examined whether *Origin*, *Time in Home*, *Age Group*, and *Sex* predicted head-tilt frequency. The model revealed significant effects of *Sex*, χ^2^(3) = 15.35, *p* = 0.002, and a marginal effect of *Age Group*, χ^2^(2) = 5.97, *p* = 0.050. No significant effects were found for *Origin* or *Time in Home* (*p* > 0.16). Examination of estimated means suggested that intact females and neutered males tended to exhibit more frequent head tilts than intact males or spayed females (see analysis below). However, the model was unstable for several *Origin* levels (large SEs and extreme confidence intervals), suggesting sparse data within some demographic combinations. As such, these results should be interpreted cautiously.

We replicated the same analysis for dog breed. The overall model revealed no significant effect of breed, χ^2^(7) = 1.35, *p* = 0.987, indicating that head-tilt frequency did not differ reliably across breeds.

### 3.2. Occurrence of Head Tilt Across Conditions

The mean number of head tilts was minimal across the first three conditions, Condition 1 (*M* = 0.019, *SD* = 0.197), Condition 2 (*M* = 0.019, *SD* = 0.139), and Condition 3 (*M* = 0.184, *SD* = 0.860), indicating that few head tilts occurred in these contexts. In contrast, Condition 4 yielded a higher frequency of head tilts (*M* = 1.097, *SD* = 1.973: see Figure 1c and Table 1).

A separate Poisson generalized linear mixed-effects model (log link) tested whether the number of head tilts changed across conditions. The model included Condition as a fixed effect and Subject as a random intercept. There was a significant main effect of Condition, χ^2^(1) = 39.22, *p* < 0.001, indicating that the frequency of head tilts increased markedly across conditions. Fixed-effects estimates (*b* = 4.05, SE = 1.55, t = 2.61, *p* = 0.009) showed that the log count of head tilts rose with each successively more social condition. Estimated marginal means confirmed a strong monotonic increase: less social conditions yielded near-zero predicted counts, while conditions in which humans communicated more overtly showed higher expected frequencies.

### 3.3. Direction of Head Tilt

Because Condition 4 was the only condition in which there were enough head tilts to be included in analysis, all further analysis of directionality is composed of data from Condition 4 only.

A chi-square test of independence was performed to determine if dogs tilted with greater frequency in either direction. Because the data were not normally distributed, the variables used in the chi-square were binomial. Nominal data were utilized to determine which direction each dog titled their head more frequently, and then directionality was binned via a 0–1 binary. First, left and right head tilts were tallied individually for each dog. Next, the direction in which each dog tilted with higher frequency according to the tally was assigned a score of 1 for that dog, while the other direction was assigned a score of 0. Both directions (left and right) were assigned a 0 score for dogs that did not tilt their heads. The frequency of 1s for each direction was then compared (right = 22, left = 15). The number of dogs who tilted their heads to the right was higher than the number who tilted to the left [X^2^ (1, 103) = 4.769, *p* = 0.029, *V*_effect_ = 0.215].

### 3.4. Direction of Head Tilt by Sex & Reproductive Status

Mixed-model analyses failed to converge due to small and unbalanced group sizes (e.g., FI = 5, MI = 10). As such, we report chi-square results, which provide a robust and transparent test of association for categorical data and yield comparable conclusions when group frequencies are the unit of analysis. To that end, a series of contingency tables and Chi-Square values were generated to assess the relationship between head tilt and sex in Condition 4 (Table 2). In the first analysis, there was a significant relationship between the occurrence of head tilts and sex [X^2^ (3, 103) = 9.366, *p* = 0.025, *V*_effect_ = 0.302]. The sex variable included four groups: neutered males (MN) (*n* = 40), spayed females (FS) (*n* = 48), intact males (MI) (*n* = 10), and intact females (FI) (*n* = 5). There was a significant difference in the occurrence of head tilt between spayed females and neutered males, X^2^ (1, 88) = 8.282, *p* = 0.004/0.024, *V*_effect_ = 0.307. The proportion of neutered males (22 out of 40, proportion = 0.55) that performed a head tilt was significantly larger than the proportion of spayed females (12 out of 48, proportion = 0.25). All other contrasts were not statistically significant (uncorrected *p*-value and Bonferroni-corrected *p*-values are reported for each contrast—e.g., *p* = uncorrected/corrected): spayed females and intact males, X^2^ (1, 58) = 0.108, *p* = 0.743/1, V = 0.043; or intact females and neutered males, X^2^ (1, 45) = 2.179, *p* = 0.140/.840, V = 0.220. There were also no statistically significant differences in head tilts between intact females and spayed females, X^2^ (1, 53) = 0.061, *p* = 0.805/1, *V* = 0.034; or between intact males and neutered males, X^2^ (1, 50) = 2.000, *p* = 0.157/.942, *V* = 0.2. Finally, there were no significant differences in head tilt between intact females and intact males, X^2^ (1, 15) = 0.170, *p* = 0.680/1, *V* = 0.107.

We further evaluated the direction of head tilt among neutered males. In Condition 4, when neutered males tilted their heads, they tilted significantly more to the right (count = 13, proportion = 0.59) than the left (count = 9, proportion = 0.41) [X^2^ (1, 40) = 5.591, *p* = 0.018, *V*_effect_ = 0.374].

No other variables were found to affect the direction of the head tilt significantly.

### 3.5. Amplitude of Head Tilt

**Figure 3:**
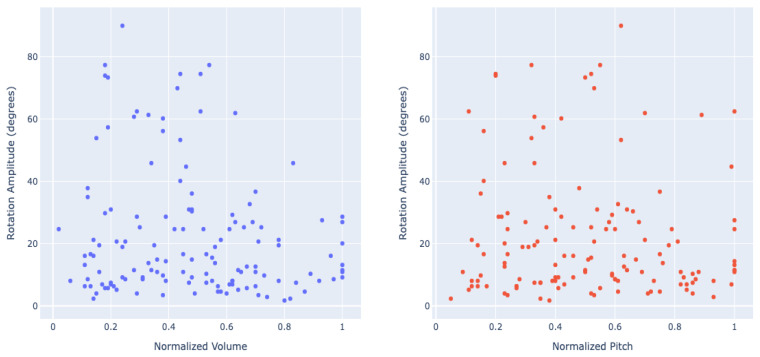
The head tilt rotation amplitude (in degrees) depending on the cause word’s normalized volume (**left**) and pitch (**right**).

To assess the effect of various parameters on each tilt’s amplitude, we considered each tilt instance separately, applying mixed-effects regression models with dogs defined as a random factor and using natural log transformation to normalize the distribution of the residuals. None of the variables (sex, breed group, age, word category, word normalized volume, and word normalized pitch) significantly predicted the tilt amplitude (*p*-values ranging from 0.08 to 0.80). Figure 3 shows the lack of dependency of head tilt amplitude (in degrees) on the audio signals normalized volume and pitch.

## 4. Discussion

Our results show that the head tilt behavior observed in dogs in response to human cues is right-lateralized, consistent with left-hemisphere lexical processing. Specifically, we found that dogs tilted their heads more to the right in response to familiar speech, and that male dogs tilted more frequently than female dogs. No significant relationship was found between head tilt parameters and semantic meaning of the communicative cues (using the four semantic categories), nor the volume or pitch of the communicative cues.

The only known prior work on the head tilt behavior of dogs reported a relationship between head tilt and the processing of meaningful stimuli [13]. In that study, individual dogs’ head tilts were consistent across experiments, but there was no consistent evidence of population-level lateralization. However, data on the direction of the head tilt were limited to six dogs of one breed. In contrast, the present study includes over 40 dogs that performed the head tilt behavior across all seven breed groups, plus mixed-breed dogs.

Work utilizing the head-*turn* behavior has suggested lateralization of processing human vocalizations in the canine brain but produced contradictory results regarding hemispheric bias for processing familiar words [11,24]. The majority of head tilts in the present study occurred in Condition 4, where owners spoke in a neutral to happy tone using words their dog was familiar with. The recorded number of head tilts in all other conditions was not significantly different from zero (including Condition 3, in which the owner was speaking for the same duration of time but utilizing unfamiliar words). Our results reveal that differences in owner communication between conditions influenced the frequency of the head-tilting behavior, consistent with the hypothesis that the head-tilt behavior is related to the processing of meaningful speech stimuli, specifically familiar words and phrases—but apparently without specific differences between particular categories of those words.

Because only Condition 4 contained enough head tilts for further statistical comparisons, directionality and amplitude differences were explored in this condition only. There were significant sex differences in the direction of head tilts between neutered males and spayed females, with neutered males tilting their heads significantly more often. In humans, the processing of semantic information occurs primarily in the left hemisphere for both sexes. However, females have been reported to show more bilateral activation during these tasks [25,26]. Likewise, in rats, more complex auditory stimuli elicit stronger right ear biases [27] indicating left hemisphere processing, and male rats exhibit significantly better tonal discrimination with the right ear than the left [28]. The results of the current study demonstrate that dogs follow this pattern as well—if males show a more obvious pattern of left-hemispheric lateralization in auditory processing, it is likely they more prevalently display an asymmetrical behavior related to it. Why unaltered males and females did not display significant differences in the present study (c.f. Supplementary Materials) remains unclear, though it should be noted that the sample size of unaltered males and females was small, an important limitation which may have contributed to the lack of significant difference seen here.

Orienting asymmetries have evidenced hemispheric biases in primates, pigs, cats, and goats, largely suggesting a left-hemispheric bias for processing species-specific, emotionally relevant auditory stimuli [29,30,31,32]. Assuming, as we do, that the head tilt behavior in dogs indexes lateralized brain processing, the results of the present study provide evidence that dogs process familiar human speech in the left hemisphere of the brain.

Furthermore, these results concur with findings from recent neuroimaging studies. Barton et al. [33] discovered leftward asymmetry of temporal cortical regions of the canine brain that are activated during human speech. The authors report that these areas of the brain are associated with communication and visual and auditory perception of humans, including the processing of speech. Using computer-generated language (focused more heavily on discrete syllables), Boros et al. [34] found a lesser activation of the left basal ganglia for structured speech in dogs in a word learning task, in addition to bilateral sensitivity of the auditory cortex to structured sequences. The basal ganglia is associated with sequence learning, among other cognitive functions, and has been linked to the differentiation of familiar and unfamiliar words [35]. These authors did not discuss sex or breed differences in the observed activation patterns.

Left-brain lateralization in dogs, in conjunction with the hemispheric lateralization patterns seen in the human literature, suggests that dogs process familiar words not just as affective cues but similarly to how humans process non-emotive language. However, we report no differences in tilting behavior (in terms of direction and amplitude) for different word categories (*Activity*, *Addressing*, *Food*, and *Toy*). No significant difference here does not mean that dogs do not differentiate between word categories, but that if they react to them by tilting their heads, there is no significant difference in this reaction.

Moreover, and from a methodological standpoint, while the AI-based analysis of head tilt amplitude did not identify significant relationships with the factors considered in this study, it still highlights the potential of a fully automated, landmark-based approach for quantifying head tilt behavior. This method offers a promising tool for future research on head tilt and related subtle behaviors such as indications of pain. While algorithms and deep learning models are still being developed and can be prone to error related to the scope and accuracy of training, the continuous behavioral signal generated by this novel approach enables the measurement of a variety of parameters (such as speed, angles or amplitudes) that are challenging to capture through traditional manual coding methods.

In one study of humans’ ability to recognize canine facial emotion, photographs of dogs in ‘Surprise’ conditions display dogs tilting their heads [36]. The ‘surprise’ emotion, and thus the head tilt, was elicited by the pop of a jack-in-the-box, suggesting that the head tilt behavior could be evoked by an interesting or surprising stimulus. The present study was focused specifically on human-canine interaction and thus did not include non-verbal and environmental auditory stimuli (e.g., whistles, squeaks, doorbells, etc.). Future research should explore the impact of these external auditory stimuli and the role of emotion in producing the head tilt.

Prior work has suggested lateralization of brain patterns in response to non-verbal vocalizations of different human emotions, with the right hemisphere being associated with fear and sadness, and the left associated with happiness [37]. Because the familiar words and phrases used in the present study were chosen by the owners and almost all had a positive connotation, we cannot rule out that the left-hemispheric bias recorded here was due to asymmetric emotional modulation of the canine brain. Future research should explore whether verbal vocalizations elicit the same hemispheric biases as these non-verbal vocalizations.

Additionally, in the current study, we investigated the effect of the familiar words’ meaning, volume, and pitch when they caused head tilt. For future studies in more controlled environments, it will also be important to consider when such words did not cause tilts versus when they did only after a few repetitions, to assess the effect more thoroughly.

Data collection was completed throughout the COVID-19 pandemic utilizing community science, meaning no in-person collection could be performed. Consequently, we were neither able to control for owners’ interactions with their dogs during in-home data collection, nor environmental (home) factors that may have facilitated or inhibited performance. For the goals of this study, data collection in a home environment might be more appropriate than in an unfamiliar environment, though replicating this study under more controlled conditions would provide a useful comparison. Additionally, we were unable to control for the amount of reading versus memorization participants undertook in Condition 3, in which dog owners were asked to repeat unfamiliar sentences to their dogs. This may have impacted the amount of eye contact participants made with their dogs in this condition. While it is our understanding that most participants were able to memorize the sentences and maintain eye contact, it is not something we were able to measure or control for. Future studies could consider controlling for this measure.

We also cannot rule out the possibility that condition sequence, in which speaking familiar words always occurred last, contributed to our results. While owners were instructed to allow at least 30 min between the filming of conditions, it is possible that the longer dogs are engaged with, the more likely they are to tilt their heads. We suggest future research reverse or randomize the order of the conditions or manipulate the duration of engagement using dog-directed speech.

In our analysis of breed groups’ differences in head tilt occurrence, there were multiple breed groups made up of fewer than ten individuals. These results should be interpreted with caution as statistical power to detect breed differences was low.

Alternative potential explanations for observed results regarding sex differences include potential differences in lateral bias between sexes. One study of lateral biases in paw preference observed significantly more male dogs being classified as right-pawed, while more females were more likely to be classified as ambilateral than left or right-pawed [38]. Our data is consistent with the idea that male dogs are right side dominant, as we saw male dogs tilting their head more frequently to the right than to the left. If female dogs are truly more ambidextrous, recording their head tilting data as binomial would have underrepresented the frequency of the behavior.

It is unclear why unaltered males and females did not display significant differences in the amount of head tilts they performed, but with only 15 unaltered dogs in the current study, this may be due to small sample size. Alternatively, the significant sex differences in the tilting response may be due to sex differences in interspecific sociality. Sommese et al. [13] suggest that the head tilt may be a sign of increased attention. If the head tilt behavior is viewed through a lens of attentiveness to human speech, neutered male dogs in the present study are presenting as more attentive and communicative in an interspecific setting, which is consistent with prior work [39,40].

## 5. Conclusions

The present study found evidence of lateralization of head tilt behavior in dogs while listening to naturalistic speech and sex differences in auditory processing that parallel sex influences in humans. Results show a left-hemispheric bias in the canine brain in the processing of familiar words and phrases spoken by humans. The dogs in this study also displayed similar patterns to humans in sex influences related to auditory processing. Males utilized the left hemisphere, while females seemed to process in a more bilateral manner.

Both our significant findings and the null results offer several directions for additional inquiry and future research that could be illuminating in terms of cognitive processing and especially language processing in dogs; interspecies interactions; and the relationship between social interaction, communication, and both intentional and unintentional behaviors.

## Figures and Tables

**Table 1:** Head tilt performance of dogs across conditions with increasing social cues, N = 103.

Condition	Min # Tilts	Max # Tilts	Mean	SD
1 (baseline)	0	2	0.019	0.197
2 (eye contact)	0	1	0.019	0.139
3 (unfamiliar speech)	0	6	0.184	0.860
4 (familiar speech)	0	10	1.097	1.973

**Table 2:** Head tilt performance of dogs in Condition 4 (high social cuing).

Dog Sex/Reproductive Status	Head Tilts in Condition 4	
	Ratio	Percent
FS	12/48	25%
MN	22/40	55%
FI	1/5	20%
MI	3/10	30%

## Data Availability

All data used to generate figures and perform analyses can be found at: https://osf.io/ypsw2/?view_only=7fbe404e20d64328933dc807b7ac7b84. Subject coding data from the parent study are available publicly via Mendeley Data: doi:10.17632/br92x9768y.1. Video: data may be available upon request in accordance with privacy considerations.

## Supplementary Materials

The following supporting information can be downloaded at: https://www.mdpi.com/article/10.3390/ani15213179/s1, Table S1: Volume and Pitch; Table S2: Dog Categorical Data; Table S3: Tilt Counts & Occurrences.
